# Improving primary care Access in Context and Theory (I-ACT trial): a theory-informed randomised cluster feasibility trial using a realist perspective

**DOI:** 10.1186/s13063-019-3299-2

**Published:** 2019-04-04

**Authors:** John A. Ford, Andy P. Jones, Geoff Wong, Garry Barton, Allan Clark, Erika Sims, Ann Marie Swart, Nick Steel

**Affiliations:** 1 Department of Public Health and Primary Care, Faculty of Medicine and Health Sciences, Norwich Medical School, University of East Anglia, Chancellors Drive, Norwich, NR4 7TJ UK; 2 Nuffield Department of Primary Care Health Sciences, University of Oxford, Oxford, UK; 3 Norwich Clinical Trials Unit, University of East Anglia, Norwich, UK

**Keywords:** Health services for the aged, Aging, Access to health care, Primary care, Vulnerable populations, Rural health services, Randomised controlled trials, Feasibility studies

## Abstract

**Background:**

Primary care access can be challenging for older, rural, socio-economically disadvantaged populations. Here we report the I-ACT cluster feasibility trial which aims to assess the feasibility of trial design and context-sensitive intervention to improve primary care access for this group and so expand existing theory.

**Methods:**

Four general practices were recruited; three randomised to intervention and one to usual care. Intervention practices received £1500, a support manual and four meetings to develop local, innovative solutions to improve the booking system and transport.

Patients aged over 64 years old and without household car access were recruited to complete questionnaires when booking an appointment or attending the surgery. Outcome measures at 6 months included: self-reported ease of booking an appointment and transport; health care use; patient activation; capability; and quality of life. A process evaluation involved observations and interviews with staff and participants.

**Results:**

Thirty-four patients were recruited (26 female, eight male, mean age 81.6 years for the intervention group and 79.4 for usual care) of 1143 invited (3% response rate). Most were ineligible because of car access. Twenty-nine participants belonged to intervention practices and five to usual care. Practice-level data was available for all participants, but participant self-reported data was unavailable for three. Fifty-six appointment questionnaires were received based on 150 appointments (37.3%).

Practices successfully designed and implemented the following context-sensitive interventions: Practice A: a stacked telephone system and promoting community transport; Practice B: signposting to community transport, appointment flexibility, mobility scooter charging point and promoting the role of receptionists; and Practice C: local taxi firm partnership and training receptionists. Practices found the process acceptable because it gave freedom, time and resource to be innovative or provided an opportunity to implement existing ideas. Data collection methods were acceptable to participants, but some found it difficult remembering to complete booking and appointment questionnaires. Expanded theory highlighted important mechanisms, such as reassurance, confidence, trust and flexibility.

**Conclusions:**

Recruiting older participants without access to a car proved challenging. Retention of participants and practices was good but only about a third of appointment questionnaires were returned. This study design may facilitate a shift from one-size-fits-all interventions to more context-sensitive interventions.

**Trial registration:**

ISRCTN18321951, Registered on 6 March 2017.

**Electronic supplementary material:**

The online version of this article (10.1186/s13063-019-3299-2) contains supplementary material, which is available to authorized users.

## Background

Good access to primary care is important for older, socio-economically disadvantaged people because they experience a greater burden of chronic disease compared to the rest of the population [1]. In the United Kingdom, primary care access is getting worse according to most General Practice Patient Survey measures, such as ease of getting through to someone at the surgery and ability to see a preferred general practitioner (GP) [2, 3]. The effect is likely to be worse for those living in rural areas, with 37% of people in rural areas having no GP surgery within 2 km compared with 1% in urban areas [4]. A previous review of access to health care found that older people, those in rural areas and socio-economically disadvantaged groups are at higher risk of poor access [5]. Whilst there are systematic reviews looking at barriers to primary care access [6, 7], little research has focussed on rural socio-economically disadvantaged older people. Systematic reviews of interventions to improve access to primary care for the wider population have called for more research examining targeted, or context-dependent, interventions [7, 8].

Using a realist perspective, we have undertaken a programme of research exploring how socio-economically disadvantaged older people in rural areas access primary care [9]. Rather than asking if an intervention works or not, realist approaches aim to explore questions such as ‘how?’, ‘why?’, ‘for whom?’, ‘in what circumstances?’ and ‘to what extent?’ [10]. To answer these context-dependent questions, a realist logic of analysis is used to build context-mechanism-outcome configurations (CMOcs) [11]. Realist approaches are well suited to intervention development because they provide a means of developing explanations and justifications for design [12]. More specifically, they provide an explanation for how and why modifying a context through an intervention should trigger a mechanism leading to an outcome.

**Fig. 1:**
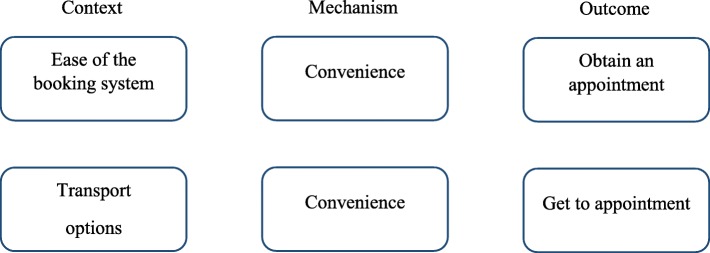
Context-mechanism-outcome configurations associated with the booking system and transport

First, we reviewed the academic literature using a realist approach, finding a range of personal, community and health care barriers that occur across a patient pathway [13]. Second, we undertook a qualitative study of older people and health professionals, identifying barriers to access, such as engaged telephone lines, availability of appointments, interactions with receptionists and transport [14]. Finally, we looked at the usefulness of structural equation modelling to explore realist theory in the English Longitudinal Study of Ageing [15]. Based on these studies, we identified the booking system and transport for those without car access as important issues suitable for intervention. They were judged to be suitable because GP surgeries could potentially influence or support them in a short time frame. The underpinning theory for these factors is described in detail elsewhere [13, 14]; however, a brief overview of the associated realist CMOcs is shown in Fig. 1. Whilst there is overlap between the concepts of *ease of booking system* and *perceived convenience*; they are different. The ease of the booking system is concerned with how simple and straightforward the process is of booking an appointment based on practice procedures and protocols, whereas convenience is more concerned with the suitability or usefulness of those processes for an individual. For example, a booking system that offers predominantly same-day appointments may be viewed as easy, but not convenient for patients without car access who need to arrange transport.

Instead of developing a broad, single, one-size-fits-all intervention to improve access for this group, we aimed to design a process to allow GP surgeries to develop and implement their own local context-sensitive interventions within a cluster feasibility study. We chose this based on discussions with local health professionals and our previous research that revealed a range of context-specific social, physical and organisational barriers to accessing services, such as bus routes and times, taxi availability and organisation, workforce and experience of the GP surgery. Consequently, within our intervention, GP surgeries were allowed to develop and implement their own service changes supported by an intervention manual, four development meetings and a £1500 grant.

We designed the I-ACT cluster feasibility trial to assess the feasibility of a trial design and context-sensitive intervention. Specifically, we aimed to (1) assess the eligibility, recruitment and retention of participants and practices, (2) assess the ability of practices to develop and implement their own service changes and acceptability of the process, (3) the acceptability of data collection methods and (4) expand the initial CMOc-based theory (Fig. 1).

## Methods

### Study design and practice recruitment

We undertook a cluster randomised controlled feasibility study. Ethical approval was obtained from the NHS North East National Research Ethics Committee (ref 16/NE/0424). We recruited four general practices in Norfolk, England, on a first-come first-served basis via the Eastern Clinical Research Network of research-active practices. Practice eligibility was: a rural practices as classified by the Health and Social Care Information Centre [16], list size of over 7000 and agreement to develop and implement service changes. Each practice was profiled using publicly available data and discussions with practice staff to describe the demographics, organisational structure and issues relating to access.

### Participant eligibility

We aimed to recruit 10 participants from each practice for data collection which was judged to be sufficient to answer the feasibility objectives. To be included, participants had to be 65 years or older and have two or more repeat prescriptions at baseline (to only include those with existing health need), 12 or fewer face-to-face GP or nurse visits over the past 12 months (to exclude frequent attenders who were less likely to have problems using the service) and no household car access. Patients were excluded if they had cognitive impairment, such that written informed consent was not possible, were unable to speak English, or did not usually book their own appointments.

### Recruitment

Practices undertook an electronic search to identify patients who met age, medication and primary care visit criteria. It was not possible to search for those without car access, so this eligibility criterion was described in the invitation letter. From the identified patients, 150 were randomly selected for invitation, providing that clinical staff judged that they were suitable (e.g. did not have significant cognitive impairment). Later, several additional strategies were introduced to increase recruitment: practices were asked to send an additional 150 patients invitations, reminder letters were sent and letters of invitations handed out by reception and in-practice pharmacy staff. If patients met the eligibility criteria and were interested, a researcher (JF) visited to obtain written informed consent and collect baseline data.

### Randomisation

All participants were recruited prior to randomisation of practices. Norwich Clinical Trials Unit undertook simple, block randomisation using sealed opaque envelopes with a ratio of three intervention practices to one usual care. Whilst it was not possible to blind participants or practices to the allocation, care was taken by the research team not to inform participants of the allocation.

### Intervention and usual care

Practices allocated to the intervention arm were asked to improve the ease of the booking system and transport options for socio-economically disadvantaged older people without access to a car. To achieve this, practices were given a support manual, containing an evidence summary and trial requirements, four development meetings with the lead researcher (JF) over a 4-week period and a grant of £1500. All practices had 2 to 3 months to develop and implement their service changes. The intervention was allowed to be targeted specifically at the group of interest or the whole practice population. Small modifications to the intervention were allowed during the trial period providing that the research team was informed. Practices were also asked to consider activity measures to assess implementation of the intervention. All development meetings were audio-recorded and transcribed, and a logic model produced. The practice allocated to usual care did not receive any of the above support.

### Quantitative patient outcomes measures

The main outcome measures, reflecting the pre-specified CMOcs and assessed using a 100-point visual analogue scale (VAS), were self-reported transport options, perceived convenience of transport, suitability of transport, ease of booking an appointment, perceived convenience of booking an appointment, and suitability of received appointment. Data was collected at baseline (researcher visit), follow-up (postal questionnaire) and every time a participant booked or attended an appointment (postal questionnaire). Other measures collected from participants at baseline and follow-up were the EQ-5D-5 L (EuroQol five dimensions questionnaire) [17], the ICECAP-O (ICEpop CAPability measure for Older people) [18], confidence and trust in their general practice and Patient Activation Measure (PAM) [19]. Patient activation is concerned with the knowledge, skills and confidence that a person has in managing their own health. For each of the above measures the difference-in-difference was calculated which is the change between baseline and 6 months for intervention versus control.

### Qualitative data collection

At the beginning of the follow-up period, two 3-h observations were undertaken at the reception area of each practice to understand the practice system and identify any important issues which may influence implementation. Written informed consent was obtained and detailed field notes taken.

At follow-up, two group interviews were undertaken at each practice to explore the development and implementation of the service changes, as well as the acceptability of the study design. Furthermore, semi-structured interviews were undertaken with eight participants across all practices to explore the acceptability of the trial design, data collection methods, implementation of the service changes and expand the initial CMOc-based theory (Fig. 1). Interviews were guided by a topic guide which included discussion of the context, mechanism and outcomes of the initial theory and emerging themes explored in subsequent interviews. Written informed consent was obtained. Interviews were audio-recorded and transcribed.

### Analysis

Descriptive analysis was used to assess the eligibility, recruitment and retention of practices and participants. To test the appropriateness of the analysis, complete case analysis of key quantitative outcomes was undertaken to compare intervention and usual care for the change between baseline and follow-up using a linear mixed model with practice included as a random effect. The intraclass correlation coefficient was estimated for each outcome, but caution is needed because of the small number of clusters [20]. Responses to the EQ-5D-5 L were converted into utility scores, a scale where zero is equal to death and one is full health, using the crosswalk mapping function [21], as recommended by the National Institute for Health and Care Excellence (NICE) [22]. Difference in primary care use between intervention and usual care for the 6 months before the trial and 6-month follow-up was assessed using a boot-strapped linear mixed model with practice as a random effect to account for the skewed distribution. All analyses were undertaken in Stata 15 [23].

Qualitative data was analysed using two different methods; thematic analysis and a realist logic of analysis. Thematic analysis was used to analyse data relating to acceptability of the intervention development, data collection methods, practice organisation, implementation of the intervention and methodological considerations for a future study. This involved familiarisation, then coding of data using NVivo [24]. Themes were then identified from the codes. A realist logic of analysis was used to expand the initial CMOcs shown in Fig. 1 [25, 26]. To do this, potential booking- or transport-related contexts associated with obtaining an appointment or getting to the surgery were identified. Then data was explored for underlying mechanisms. Only CMOcs relating to the booking system and obtaining an appointment or transport and getting to the appointment were identified.

Due to the size of the study, we did not undertake a full economic evaluation but did aim to identify the total cost of the intervention and the associated main cost drivers. An NHS perspective was taken and 2016/2017 costs in British pounds used throughout. Practices were asked to record on a web-based form any expenditure or time spent on their intervention. These were categorised into one-off costs (e.g. development costs) or recurrent costs (e.g. ongoing costs of the intervention) and out-of-pocket costs (e.g. external training fees) or staff time. Any costs that were no longer incurred as a result of the intervention, e.g. previous line rental fees, were also noted. An equivalent annual cost per patient was estimated based on a 3-year useful lifetime and discounting of 3.5% for each cost [27]. The number of patients per practice who were older, socio-economically disadvantaged and without access to a car per practice were estimated using published sources [28–30].

Health care utilisation data was collected from electronic patient records by the lead researcher (JF) for 6 months before and during follow-up. Data collected included: number of GPs, nurse and health care assistant appointments (split by surgery, home or telephone); accident and emergency attendances; hospital admissions (split by emergency or elective); out-of-hours primary care contact; and ambulance use (spilt by hear and treat, see and treat or convey). Primary care costs were based on Personal Social Services Research Unit costs [31] and secondary care on NHS Reference costs [32]. Unit costs are shown in Additional file 1: Table S1.

## Results

### Recruitment and completion rates

**Fig. 2:**
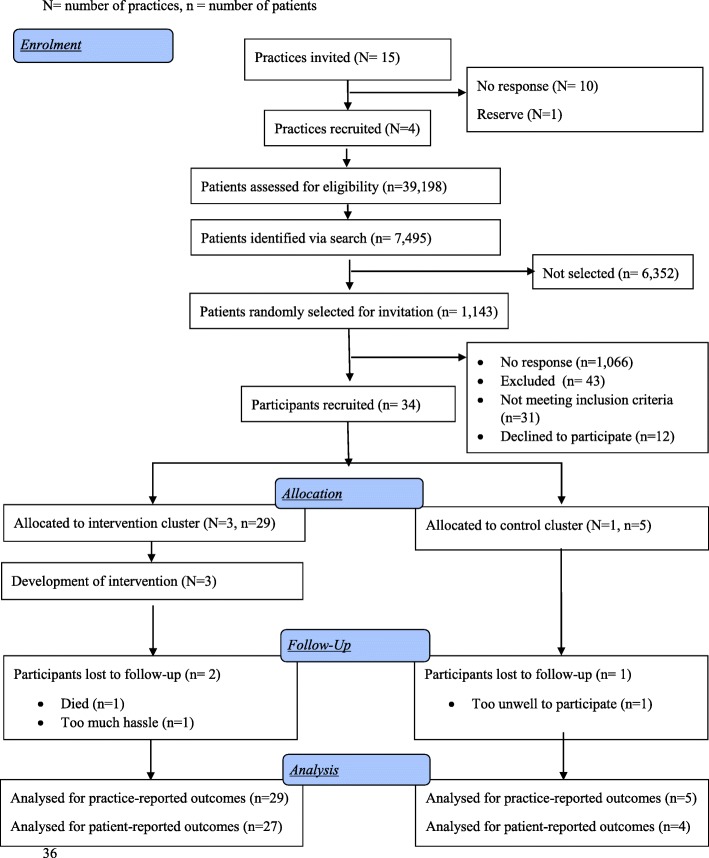
Consort flow diagram. *N* = number of practices, *n* = number of patients

Fifteen primary care practices were invited, five expressed interest and four were recruited (Fig. 2). In total 1143 participants were invited from a target of 1200 because not all practices sent all 150 additional letters (Additional file 2: Table S2). Thirty-four participants were recruited (3% response rate) between April and October 2017. Twenty-nine participants were registered at intervention practices and five at the usual care practice. The response rate varied between practices (Additional file 2: Table S2) with a range of 5.4 (Practice A with 336 approached and 18 recruited) to 1.7% (Practice C with 238 approached and four recruited). Three participants did not complete follow-up by end of study in June 2018 (91% completion rate), two of which were from Practice B in the intervention arm and one of which was from the usual care. Fifty-six appointment questionnaires were received based on 150 appointments (37.3%).

### Baseline characteristics of patients

The mean age of participants in the intervention was 81.7 years and in usual care 79.4 (Table 1). All participants were white and most were female. Fifty-nine percent of participants in the intervention practices had completed their education before the age of 16 years, compared to 20% in usual care. Participants in Practices C and D lived furthest from the surgery and those in Practice A closest. More participants in the intervention arm walked to the surgery or took taxis and more people in the usual care arm relied on lifts from friends or family. All participants in Practices C and D would definitely recommend the surgery compared to 56% in Practice A.

**Table 1:** Baseline characteristics of included participants

Variable	Practice			Intervention (*n* = 29)	Usual care (*n* = 5)
	A (*n* = 18)	B (*n* = 7)	C (*n* = 4)		
Age, mean (SD)	81.00 (8.66)	84.29 (8.16)	80.00 (4.16)	81.66 (8.01)	79.40 (8.08)
Gender					
Female	12 (67%)	7 (100%)	3 (75%)	22 (76%)	4 (80%)
Ethnicity					
White – British	18 (100%)	6 (86%)	4 (100%)	28 (97%)	4 (80%)
White – other	0 (0%)	1 (14%)	0 (0%)	1 (3%)	1 (20%)
Age at completion of education					
Before 15 years old	4 (22%)	2 (29%)	2 (50%)	8 (28%)	0 (0%)
15 or 16 years old	6 (33%)	1 (14%)	2 (50%)	9 (31%)	1 (20%)
17 to 20 years old	5 (28%)	2 (29%)	0 (0%)	7 (24%)	2 (40%)
After 21 years old	3 (17%)	2 (29%)	0 (0%)	5 (17%)	2 (40%)
Revised Family Resources Survey					
Finances do not impair standard of living in any measures	17 (94%)	7 (100%)	4 (100%)	28 (97%)	5 (100%)
Finances impair standard of living in 1 or more measures	1 (6%)	0 (0%)	0 (0%)	1 (3%)	0 (0%)
Lubben Social Network Scale 6-item, mean (SD)	14.44 (6.05)	14.00 (6.22)	16.00 (6.27)	14.55 (5.93)	15.40 (6.19)
Activities of Daily Living, mean (SD)	1.06 (1.85)	1.00 (1.15)	0.50 (1.00)	0.96 (1.57)	0.80 (1.10)
Instrumental Activities of Daily Living, mean (SD)	0.41 (0.71)	0.57 (0.79)	0.50 (1.00)	0.46 (0.74)	1.00 (1.00)
Distance from home to GP surgery, mean (SD)	0.77 (0.29)	2.09 (2.17)	3.95 (2.34)	1.56 (1.74)	3.58 (2.45)
How do you usually get to the GP surgery?					
Walk	7 (32%)	3 (38%)	1 (14%)	11 (30%)	0 (0%)
Public transport	3 (14%)	1 (13%)	2 (29%)	6 (16%)	2 (25%)
Taxi	10 (145%)	1 (13%)	2 (29%)	13 (35%)	1 (13%)
Community transport	0 (0%)	1 (13%)	1 (14%)	2 (5%)	0 (0%)
Lift from a friend or relative	0 (0%)	0 (0%)	1 (14%)	1 (3%)	3 (38%)
Home visits only	0 (0%)	2 (25%)	0 (0%)	2 (5%)	1 (13%)
Other	2 (9%)	0 (0%)	0 (0%)	2 (5%)	1 (13%)
Recommend surgery					
No, definitely not	0 (0%)	1 (14%)	0 (0%)	1 (3%)	0 (0%)
Not sure	1 (6%)	0 (0%)	0 (0%)	1 (3%)	0 (0%)
Yes, probably	7 (39%)	1 (14%)	0 (0%)	8 (28%)	0 (0%)
Yes, definitely	10 (56%)	5 (71%)	4 (100%)	19 (66%)	5 (100%)

*GP* general practice, *SD* standard deviation

### Baseline characteristics of practices and profiles

Practice A had the highest practice population but the smallest catchment area (Additional file 2: Table S2). Based on the GP Patient Survey results, Practice A had the lowest access scores compared to other practices. All practices had either a dispensary or a co-located pharmacy.

Based on the observations at the start of the trial, Practice A had the busiest reception area, with some patients attending the surgery in person because of engaged telephone lines and pressures on the appointment system. Practice B had an existing signposting process, where patients were asked about their health problem and directed to the most appropriate service, meaning that receptionists spent more time on the telephone with each patient but were more deliberate in booking appointments. Practice C reported difficulty with access to taxis, especially during busy school times. The practice also did not have any nurse specialists, and, therefore, most appointments were scheduled with GPs, sometimes for issues which could have been dealt with by a different team member. The usual care arm, Practice D, had a policy of releasing appointments at 8 a.m. and 12 noon and on one of the observation days an afternoon GP appointment remained unfilled, which the staff reported happened occasionally.

### Intervention development by practices

The logic model for each practice intervention is shown in Additional file 3: Table S3. Practice A decided to implement a call-stacking system, where calls are placed in a queue, and aimed to develop closer links with a community transport provider (Table 2). The call stacking system could hold up to 100 callers using a Cloud-based N3 Internet connection (high-speed broadband connection used in the NHS). Practice B incorporated community transport into their signposting, allowed more flexibility for receptionists to move appointments based on bus times, installed a charging point for mobility scooters and promoted the role of receptionists through a practice leaflet. Signposting is widely used in the NHS to direct patients who contact primary care to the most appropriate service, but it does not often include community transport providers which. The charging point was a designated area in reception for patients to park mobility scooters for charging. Practice C worked with a local taxi firm to develop a priority hour with corresponding taxi appointment slot and had three external training sessions for receptionists about local services and signposting. The taxi slots meant that everyday there was an embargoed appointment reserved for patients who relied on taxis. If the slot had not been booked by the day of the appointment, it was released for any patient.

**Table 2:** Summary of interventions developed

Practice	Intervention
A	• Telephone system to stack calls • Linking with, and promoting, community transport
B	• Signposting to community transport • Flexible appointment around bus times • Charging for mobility scooters • Promoting the role of medical receptionists
C	• Working with local taxi firm and creating a taxi appointment slot • Three external receptionist training sessions about local services and signposting/customer services

Practices A and C had out of pocket expenditure (£2262 and £930) for the intervention, whereas Practice B had only staff time costs (Table 3). The annual equivalent cost over a three year lifetime, per older, socio-economically disadvantaged patient without car access (Table 4) for out-of-pocket costs, was lowest in Practice A (− £13) and highest in Practice C (£2) and staff time costs were lowest in Practice A (£0) and highest in Practice C (£63). Practice A had a monthly cost saving from the new system because of cheaper call rates and the high cost in Practice C reflects the signposting of every call by the receptionists.

**Table 3:** Total cost of intervention over 6-month trial period for each practice

	One-off costs			Recurrent costs			Total costs (one-off and recurrent)		
	Practice			Practice			Practice		
	A	B	C	A	B	C	A	B	C
Out-of-pocket costs	£4680	£0	£930	− £2418^a^	£0	£0	£2262	£0	£930
Staff time	£112	£134	£1322	£0	£475	£1329	£112	£610	£2651
Total costs	£4792	£134	£2252	− £2418^a^	£475	£1329	£2374	£610	£3581

^a^Practice A had a monthly cost saving from the new system because of cheaper call rates compared to their previous contract

**Table 4:** Equivalent annual cost per older, socio-disadvantaged older patient without access to a car for each intervention practice

	One-off costs			Recurrent costs			Total costs (one-off and recurrent)		
	Practice			Practice			Practice		
	A	B	C	A	B	C	A	B	C
Out of pocket costs	£6	£0	£2	− £19	£0	£0	− £13	£0	£2
Staff time	£0	£0	£3	£0	£5	£60	£0	£5	£63
Total costs	£6	£0	£4	− £19	£5	£60	− £12	£5	£65

Assumes a 3-year useful lifetime and 3.5% annual discounting

Based on analysis of the intervention development meetings and group interviews with practice staff, the interventions developed ranged from existing ideas which practices were already considering implementing (e.g. a call-stacking telephone system) to new ideas stimulated by the freedom, time and resource to be innovative (e.g. taxi slots). The process meant that all practices had ownership of their intervention. Practices reported liking the short time scales and deadlines imposed by the intervention development process because of the momentum. All practices found it easier to develop interventions related to the booking system, rather than transport.

### Intervention implementation and usual care arm

Practice A successfully implemented the call-stacking system and whilst they advertised community transport in the reception area, they were unable to establish closer links because of a change in personnel at the community transport provider. Practice B successfully implemented their intervention and at 6 months receptionists reported signposting to community transport and changing appointments for bus timetables on average once a week. Practice C introduced the taxi slots and had one external training event before the trial began and the two during the 6-month follow-up. Activity measures proposed by the intervention practices to assess implementation were not sufficiently robust to interpret. Practice D installed a new telephone system during the follow-up period because their previous contact had expired. The new system had call stacking as a feature, but it was primarily a financial decision and the practice did not perceive a problem with engaged telephone lines.

### Impact of intervention

Staff in Practice A reported fewer complaints and patients visiting the surgery to make an appointment because of engaged telephone lines after the implementation of call stacking. Participants generally liked the call-stacking system because it gave them information about the likely wait and more confidence that the call would be answered. However, both staff and participants stated that more receptionists were needed to answer the calls; for example, 33 patients were queued on one occasion. According to staff in Practices B and C, signposting improved the availability of appointments and GPs liked a reason for the consultation being added to the electronic appointment because this helped identification of emergencies and planning. Some participants liked signposting because they felt it enabled the receptionists to prioritise, others had grown to accept it, whilst others did not perceive it as the receptionists’ role. The only participant in Practice B who used a mobility scooter reported not requiring the charging point during the study period, but said that it gave her reassurance. Staff in Practice C reported that the training improved their knowledge about local services, confidence in signposting and dealing with difficult patients. Receptionists reported only rarely using the taxi slots and no participants reported using them.

Tables 5 and 6 show the monthly change and difference in difference for each CMOc. Caution is needed interpreting the differences because of the small numbers of observations, especially in the usual care arm. Ease of booking an appointment scores improved most in Practice B and C, compared to Practice A, which did not improve, and usual care. However, the convenience of booking an appointment increased most in Practice B and usual care with a decrease in Practice C. Transport measures improved in all practices except for Practice C where transport options and ability to get suitable transport decreased.

**Table 5:** Baseline, follow-up and monthly mean change in visual analogues (score from 0 to 100) for the booking context mechanism outcome configuration

	Ease of booking an appointment					Convenience of booking appointment					Ability to book appointment				
	Practice			Interventio*n* total (*n* = 27)	Usual care (*n* = 3)	Practice			Interventio*n* total (*n* = 27)	Usual care (*n* = 3)	Practice			Interventio*n* total (*n* = 27)	Usual care (*n* = 3)
	A (*n* = 18)	B (*n* = 5)	C (*n* = 4)			A (*n* = 18)	B (*n* = 5)	C (*n* = 4)			A (*n* = 18)	B (*n* = 5)	C (*n* = 4)		
Pre-intervention, mean (SD)	52.0 (26.1)	54.3 (26.6)	56.8 (44.5)	53.2 (28.0)	65.4 (20.1)	58.0 (33.1)	65.8 (31.3)	79.3 (25.1)	62.7 (31.6)	64.8 (35.3)	61.4 (26.8)	58.3 (30.3)	74.6 (22.5)	62.6 (26.6)	75.8 (12.7)
Change from baseline to															
month 1, mean (SD)	24.4 (17.8)	NA	38.8 (68.2)	28.5 (32.2)	NA	23.4 (19.3)	NA	20.5 (29.0)	22.6 (19.8)	NA	30.6 (17.6)	NA	12.3 (18.0)	24.5 (18.4)	NA
month 2, mean (SD)	22.4 (27.6)	43.0 (NA)	NA	25.3 (26.4)	− 44.0 (NA)	20.6 (21.8)	19.0 (NA)	NA	20.4 (19.9)	− 1.0 (NA)	30.9 (12.1)	20.0 (NA)	NA	29.4 (11.8)	− 64.0 (NA)
month 3, mean (SD)	− 10.8 (22.6)	24.8 (23.7)	87.3 (NA)	18.5 (39.9)	NA	6.9 (50.2)	1.5 (11.8)	− 2.5 (NA)	3.7 (33.7)	NA	14.0 (55.4)	2.3 (29.7)	1.5 (NA)	8.1 (40.1)	NA
month 4, mean (SD)	NA	NA	NA	NA	NA	NA	NA	NA	NA	NA	NA	NA	NA	NA	NA
month 5, mean (SD)	14.8 (49.4)	34.0 (NA)	NA	18.0 (44.9)	NA	21.4 (61.4)	− 9.0 (NA)	NA	16.3 (56.3)	NA	19.0 (50.8)	34.0 (NA)	NA	21.5 (45.8)	NA
month 6, mean (SD)	14.7 (25.8)	38.0 (39.0)	87.8 (NA)	24.0 (33.0)	− 4.0 (34.1)	8.7 (30.5)	20.7 (41.7)	− 0.2 (NA)	10.9 (31.6)	24.0 (16.8)	24.2 (34.0)	37.1 (40.0)	2.7 (NA)	26.0 (34.1)	− 17.0 (47.0)
final follow-up, mean (SD)	− 1.9 (26.8)	25.1 (12.7)	28.3 (52.5)	7.6 (31.6)	6.0 (39.4)	1.2 (23.4)	11.3 (15.9)	− 13.3 (21.4)	0.9 (22.4)	22.0 (37.0)	6.1 (31.6)	9.1 (5.9)	− 7.9 (7.4)	4.3 (26.6)	2.0 (19.7)
Difference in difference without clustering (95% confidence interval)	1.6 (− 34.0 to 37.2)					− 21.1 (− 47.7 to 5.5)					2.3 (− 26.3 to 30.9)				
Difference in difference adjusted for clustering (95% confidence interval)	7.9 (− 38.3 to 54.1)					− 21.1 (− 46.6 to 4.4)					2.3 (− 25.0 to 29.7)				

*NA* not applicable, *SD* standard deviation

**Table 6:** Baseline, follow-up and monthly mean change in visual analogues (score from 0 to 100) for the transport context mechanism outcome configuration

	Transport options					Convenience of transport					Ability to get suitable transport				
	Practice			Interventio*n* total (*n* = 27)	Usual care (*n* = 3)	Practice			Interventio*n* total (*n* = 27)	Usual care (*n* = 3)	Practice			Interventio*n* total (*n* = 27)	Usual care (*n* = 3)
	A (*n* = 18)	B (*n* = 5)	C (*n* = 4)			A (*n* = 18)	B (*n* = 5)	C (*n* = 4)			A (*n* = 18)	B (*n* = 5)	C (*n* = 4)		
Pre-intervention, mean (SD)	65.5 (27.5)	70.1 (33.7)	71.6 (27.7)	67.5 (28.1)	47.8 (33.4)	69.3 (30.1)	66.9 (38.5)	77.5 (30.5)	69.8 (31.2)	45.4 (30.3)	77.7 (19.7)	61.7 (34.2)	83.1 (22.8)	75.0 (24.1)	75.3 (28.4)
Change from baseline to															
month 1, mean (SD)	2.7 (7.6)	NA	− 38.5 (5.7)	− 11.0 (22.2)	NA	− 1.1 (3.3)	0.0 (NA)	− 45.0 (NA)	− 9.7 (19.9)	NA	14.9 (15.0)	33.0 (NA)	− 91.0 (NA)	− 2.7 (51.1)	NA
month 2, mean (SD)	5.0 (9.5)	− 13.0 (NA)	NA	2.4 (11.0)	NA	9.9 (21.4)	NA	NA	9.9 (21.4)	NA	16.8 (16.4)	NA	NA	16.8 (16.4)	NA
month 3, mean (SD)	0.2 (21.5)	− 4.2 (12.8)	− 42.3 (NA)	− 7.8 (21.1)	NA	2.8 (1.8)	3.5 (NA)	NA	3.0 (1.3)	7.5 (NA)	− 3.5 (14.5)	NA	NA	− 3.5 (14.5)	8.0 (NA)
month 4, mean (SD)	NA	NA	NA	NA	NA	19.9 (21.4)	− 2.0 (NA)	NA	15.5 (21.0)	7.5 (NA)	NA	NA	NA	NA	NA
month 5, mean (SD)	− 15.0 (20.2)	− 14.0 (NA)	NA	− 14.8 (18.0)	NA	20.0 (21.6)	4.3 (2.5)	− 1.0 (NA)	12.5 (18.1)	30.0 (26.2)	− 32.5 (43.0)	4.5 (3.5)	− 0.5 (NA)	− 17.4 (35.9)	24.3 (24.4)
month 6, mean (SD)	1.0 (35.1)	− 6.0 (18.2)	− 42.5 (NA)	− 2.8 (32.8)	7.3 (14.6)	8.6 (22.1)	5.8 (4.5)	58.0 (NA)	11.7 (23.6)	40.8 (38.7)	7.5 (38.5)	2.0 (NA)	42.0 (NA)	9.9 (36.3)	17.1 (18.7)
Final follow-up, mean (SD)	6.7 (20.6)	6.1 (15.8)	− 9.8 (43.1)	4.6 (22.7)	13.5 (14.3)	4.5 (20.3)	11.4 (21.8)	8.3 (32.3)	6.3 (21.2)	1.0 (12.4)	2.5 (29.3)	20.5 (28.5)	− 18.1 (49.4)	2.1 (33.2)	6.6 (21.6)
Difference in difference without clustering (95% confidence interval)	− 8.9 (− 33.1 to 15.4)					5.3 (− 17.2 to 27.7)					− 4.5 (− 40.1 to 31.0)				
Difference in difference adjusted for clustering (95% confidence interval)	− 8.9 (− 32.1 to 14.3)					5.3 (− 16.2 to 26.7)					− 4.5 (− 38.5 to 29.4)				

*NA* not applicable, *SD* standard deviation

Table 7 shows the difference in difference for quality of life, capability and patient activation, Again, caution is needed in interpretation because of the small numbers. Quality of life decreased in all intervention practices but increased in the usual care practice. There was little difference in ICECAP-O scores between intervention and usual care practices. There was a mean drop of 21 points in PAM scores in the usual care arm, but little change in the intervention practices. Intraclass correlation coefficients are shown in Additional file 4: Table S4. Self-reported quality of care was recorded at baseline and follow-up but due to small numbers the data was difficult to interpret (Additional file 5: Table S5).

**Table 7:** Mean change between baseline and follow-up in quality of life, capability and patient activation for individual practices, intervention combined and usual care

	Practice			Intervention total (*n* = 27)	Usual care (*n* = 4)	Difference in difference (95% CI)
	A (*n* = 18)	B (*n* = 5)	C (*n* = 4)			
EQ-5D-5 L, mean (SD)						
Baseline	0.75 (0.20)	0.77 (0.16)	0.88 (0.09)	0.77 (0.18)	0.67 (0.37)	− 0.17 (− 0.33 to − 0.02)
Follow-up	0.64 (0.23)	0.72 (0.16)	0.83 (0.08)	0.68 (0.21)	0.75 (0.32)	
Difference	− 0.11 (0.14)	− 0.05 (0.12)	− 0.05 (0.07)	− 0.09 (0.13)	0.09 (0.08)	
ICECAP-O, mean (SD)						
Baseline	0.81 (0.14)	0.81 (0.10)	0.86 (0.11)	0.81 (0.13)	0.88 (0.15)	− 0.01 (− 0.14 to 0.11)
Follow-up	0.73 (0.14)	0.77 (0.10)	0.86 (0.11)	0.76 (0.14)	0.84 (0.18)	
Difference	− 0.08 (0.11)	− 0.04 (0.08)	0.01 (0.05)	− 0.06 (0.10)	− 0.04 (0.04)	
PAM, mean (SD)						
Baseline	62.17 (13.40)	56.08 (14.67)	48.27 (6.79)	59.39 (13.52)	79.43 (19.76)	22.88 (5.92 to 39.83)
Follow-up	60.47 (12.80)	64.86 (14.40)	48.73 (5.95)	59.96 (12.95)	58.10 (15.80)	
Difference	− 1.69 (11.58)	8.78 (12.16)	0.47 (2.43)	0.57 (11.51)	− 21.33 (21.20)	

*CI* confidence interval, *EQ-5D-5* L EuroQol five dimensions questionnaire, *ICECAP-O*  ICEpop CAPability measure for Older people, *PAM* Patient Activation Measure, *SD* standard deviation

There was little difference in primary care contact between intervention and usual care in the 6 months prior to the trial compared to follow-up (Table 8). The main resource cost drivers were unplanned hospital admissions, GP surgery visits and accident and emergency visits (Additional file 6: Table S6), but the small numbers and wide variation make it difficult to draw conclusions.

**Table 8:** Mean change in the number of primary care contacts for 6 months before follow-up and during follow-up for individual practices, intervention combined and usual care

	Practice			Intervention total (*n* = 29)	Usual care (*n* = 5)
	A (*n* = 18)	B (*n* = 7)	C (*n* = 4)		
Any primary care contact^a^					
Previous 6 months, median (IQR)	3.0 (2.0, 8.0)	2.0 (0.0, 4.0)	3.5 (2.0, 11.0)	3.0 (2.0, 5.0)	3.0 (3.0, 8.0)
Follow-up 6 months, median (IQR)	3.5 (1.0, 7.0)	3.0 (2.0, 7.0)	2.0 (1.0, 13.0)	3.0 (2.0, 7.0)	3.0 (0.0, 7.0)
Change between two periods, median (IQR)	0.0 (− 1.0, 4.0)	2.0 (0.0, 5.0)	0.0 (− 2.5, 3.5)	0.0 (− 1.0, 4.0)	− 1.0 (− 1.0, 0.0)
Difference in difference (95%CI)	0.49 (− 2.36 to 3.35)				

*IQR* interquartile range

^a^include surgery appointment, telephone appointment or home visit by GP, nurse or health care assistant

No complaints were received from any practice about the interventions.

### Staff and participant views on future study design

Intervention practice staff reported that it may have been useful to learn from other practices. Fifteen hundred pounds was viewed as adequate, but not enough for wider transformation. The support manual provided to practices, including evidence summary and examples of possible interventions, was rarely used. At the end of the trial, all intervention practices reported that they were thinking about further developing their interventions (e.g. installing a monitor in reception area to show the number of calls queued), but none had modified the intervention during follow-up. All participants interviewed found the questionnaires quick and easy to complete, although some found it difficult remembering to complete them.

### Expanding the initial CMOc

Emerging CMOcs, based on the participant and staff interviews, are shown in Table 9. Important mechanisms were convenience, reassurance, confidence, trust and flexibility. Some CMOcs were directly related to the interventions developed. For example, when patients are acknowledged and given information when calling, such as through call stacking (context), this triggers the mechanism of increased confidence of speaking to a receptionists, leading to the outcome of increased likelihood of getting an appointment. Whereas others were not directly related to the interventions; for example, if a GP or nurse tells a patient that they need an appointment, this triggers efficient action leading to an increased likelihood of booking an appointment.

**Table 9:** Expanded context mechanism and outcome configurations

Context	Mechanism	Outcome
Booking system		
Acknowledgement and information (e.g. being held in a queue)	Confidence	Ability to book an appointment
Knowledgeable and empowered receptionists (e.g. effectively signposting with backing from GPs and senior staff)	Trust	
Acceptance of booking system	Engagement	
Primary care staff authorisation of future appointment	Efficient action	
Available appointments with usual GP	Reassurance and continuity	
Short wait on telephone	Convenience	
Transport options		
Resources to support transport at surgery (e.g. charging point or taxi booking service)	Reassurance	Ability to get to the surgery
Friends, family or neighbours with access to a car	Flexibility	
Familiar transport routine (e.g. using a the same taxi firm or bus to travel to the doctors combined with shopping)	Efficiency	
Financial resources and willingness to pay for a taxi	Autonomy	
Suitable public transport routes and times	Convenience	
Ability to walk to surgery	Reassurance	

## Discussion

Practices were able to successfully design and implement their own context-sensitive service changes based on development meetings, a £1500 grant and, to a lesser extent, the use of a support manual. They found the process acceptable because it gave them the freedom, time and resource to be innovative or provided an opportunity to implement existing ideas. Recruiting older participants without car access proved challenging, with only a 3% response rate. Retention of participants and practices was good but only about a third of appointment questionnaires were returned. Refined theory highlighted important contexts and mechanisms related to access and the interventions.

### Strengths and limitations

The overarching realist programme theory (Fig. 1) and standardised support package given to intervention practices provided a base from which practices could develop their own service changes. It enabled a comparison between intervention and usual care, whilst also allowing for an understanding of the relative impact of each individual intervention. Profiling and observations were undertaken to understand the characteristics and dynamics of practices. We believe this increases the utility of evidence produced because practitioners can understand what solutions were developed for particular issues and their relative impact. Not only was the trial driven by realist theory, it also expanded the initial CMOcs to provide a clearer understanding of access to primary care for this group. Therefore, whilst some participants may have found it difficult to differentiate between ease and convenience, our revised theory has proposed improved CMOcs. This was an experimental trial design, drawing on realist approaches, and, therefore, the study did not entirely align with standard feasibility procedures. For example, we did not have a primary outcome because we sought to explain the multiple effects of this complex intervention. This is supported by Medical Research Council (MRC) guidance which states that whilst a single primary outcome and small number of secondary outcomes to evaluate complex interventions is the most straightforward from a statistical point of view, this may not provide an adequate assessment of success [33].

Whilst retention was good, the recruitment rate was poor primarily because of the eligibility criteria requiring no car access. Due to the recruitment strategy, it was not possible to estimate the eligible population without access to a car. Furthermore, the proportion of appointment questionnaires returned compared to appointments was 37.3%, although this figure may be underestimated because of joint appointments. Practice A was not able to implement closer links with the community transport provider, but other proposed changes were implemented. Implementation activity measures were not sufficiently robust, but qualitative data on implementation was collected during the end-of-study interviews.

### Implications for a definitive trial

Future studies should consider alternative means of collecting data, rather than recruiting individual patients which proved difficult. Intervention practices found it easier to develop interventions relating to the booking system rather than transport, suggesting that wider community and stakeholder action is needed to improve transport. Practices A and C used some of the £1500 grant for out-of-pocket expenditure, whereas Practice B only had staff time costs. Whilst it could be argued that achieving the outcome at the lowest cost is desirable, practices may have been more innovative if the grant was limited to out of pocket expenditure.

After a few months, it became clear that the taxi slots were not being used, but the practice continued until completion despite ideas for improvement. Future studies may consider a review period during the trial to allow practices an opportunity to make small modifications with any significant changes incorporated into the analysis plan.

### Comparison with other studies

Adaptive intervention designs have been used for individual patient management [34–36], but less often for complex interventions. The RADiP trial randomised 795 dental practices in Scotland to either an audit and feedback intervention to improve antibiotic prescribing or control [37]. The intervention practices were then able to develop their own local solutions to improve prescribing habits. The authors found a statistically significant improvement in antibiotic prescribing. Our study has similarities because it allowed intervention practices to develop their own solutions, but for an arguably more complex issue.

Use of realist approaches within a trial have been debated [38–43]. Bonell and colleagues proposed a ‘realist RCT’ [38], subsequently publishing an example of a whole-school intervention aimed at reducing aggression and bullying [39]. The authors refined their initial realist theory through a process evaluation before the collection of quantitative follow-up data, which was analysed using mediation and moderation analyses. However, the design was criticised because the nature of the mechanisms, method of statistical analysis and inconsistent philosophical paradigms [40, 41]. Here we do not propose a ‘realist trial’, but rather use realist theory and principles to design a trial to produce more useful evidence for decision-makers. Fletcher and colleagues argue that using realist principles across the phases of the MRC Framework [44] will facilitate better evaluation of complex interventions [12].

Two key linked considerations in the evaluation of complex interventions are standardisation [45] and generalisability [46]. Previous MRC guidance on complex interventions stated that trials should ‘consistently provide as close to the same intervention as possible’ by ‘standardising the content and delivery of the intervention’ in every site [47]. However, the 2008 guidance [33] acknowledges that complex interventions may change and some interventions are specifically designed to adapt to local circumstances [48, 49]. A rigid, standardised intervention which aims to be the same in every setting may subsequently reduce the generalisability because, in real life, practitioners modify intervention to complement existing practices, policies and services. Our trial design uses middle-range [50], theory of commonly found mechanisms and, hence, may be more transferable, increasing generalisability.

### Implications for research and policy

Practices were successfully able to design and implement context-sensitive interventions and found the process liberating and empowering. Researchers and policy-makers should consider giving general practices opportunities to develop innovative, context-sensitive solutions for local problems, rather than dictating ‘one-size-fits all’ interventions. However, the process needs managed with dedicated time, resource and willingness from practices. A future trial should test the effectiveness of a support package, including financial support and development meetings, to help practices develop their own service changes. Furthermore, the support manual could be shortened, limiting it to development requirements and examples. A review after 6 weeks and, if necessary modification, may help practices to optimize their service changes. It is likely that further feasibility testing would be needed before a definitive trial.

Research methods need to evolve to generate more useful evidence for decision-makers. Katikireddi and colleagues found that most policy initiatives were likely to be ineffective or lacked the evidence to establish effectiveness [51]. This is unsurprising since only one in four policy-makers report using review articles and evidence summaries or academic journals as a source of information [52]; a finding supported by other researchers [53–55]. Here we present a study design, based on theory and a standardised, evidence-based support package that also provides context-sensitive exemplar interventions of the operationalisation of the theory. We believe that this design is more likely to produce useful evidence for decision-makers because it does not assume that ‘one-size-fits all’ or judge success based on a single primary outcome, but rather proposes local solutions for local problems explaining their likely effects.

## Conclusion

Recruiting older participants without access to a car proved challenging, but retention was good. Practices were able to successfully design and implement their own context-sensitive service changes, giving them the freedom, time and resource to be innovative or provided an opportunity to implement existing ideas. It is hoped this study design may facilitate a shift from one-size-fits-all approaches to solutions which are more context-sensitive and facilitate a greater theoretical understanding of the problem and intervention.

## Additional files

Additional file 1: **Table S1.** Unit costs [31, 32, 56]. (DOCX 15 kb)

Additional file 2: **Table S2.** Characteristics of included practices. (DOCX 15 kb)

Additional file 3: **Table S3.** Logic model for intervention practices. (DOCX 16 kb)

Additional file 4: **Table S4.** Intraclass correlation coefficient. (DOCX 14 kb)

Additional file 5: **Table S5.** Quality of care at baseline and follow-up for those with complete data. (DOCX 26 kb)

Additional file 6: **Table S6.** Resource use activity and associated costs. (DOCX 23 kb)
