# Supplementary material for: Improving primary care Access in Context and Theory (I-ACT trial): a theory-informed randomised cluster feasibility trial using a realist perspective
Source: Trials. 2019 Apr 4;20:193. doi: 10.1186/s13063-019-3299-2 (PMC6449944; doi:10.1186/s13063-019-3299-2)
Supplement: Supplementary file 1 — Table S1. Unit costs [31, 32, 56]. (DOCX 15 kb) [file 13063_2019_3299_MOESM1_ESM.docx]

**Table S1** Unit costs.

| Activity | Unit Cost | Source |
| --- | --- | --- |
| Hourly rate of receptionist | £24.50 | PSSRU 2017 – hourly rate for Agenda for Change Band 2 staff extrapolated from average proportion of hourly rate to salary for Band 4, 5 and 6 |
| Hourly rate of practice manager | £30.50 | PSSRU 2017 – hourly rate for Agenda for Change Band 4 staff |
| Hourly rate for dispensary staff | £27.31 | PSSRU 2017 – hourly rate for Agenda for Change Band 3 staff extrapolated from average proportion of hourly rate to salary for Band 4, 5 and 6 |
| Signposting a call | £0.20 | Personal correspondence from Practice C that it takes on average 30 second of receptionist’s time per call to signpost |
| Signposting to community transport | £0.80 |  |
| Change embargoed slot to suit bus timetable | £0.40 |  |
| GP surgery consultation | £31 | PSSRU 2017 |
| GP home visit | £65.38 | Patient contact and travel time based on PSSRU 2015 and hourly rate PSSRU 2017 |
| GP telephone consultation | £24.26 | Time based on PSSRU 2015 and hourly rate PSSRU 2017 |
| Nurse surgery appointment | £12.47 | Time and hourly cost of direct patient care based on 2015 PSSRU inflated to 2017 costs based on PSSRU inflation indices |
| Nurse telephone consultation | £4.99 | Time and hourly cost of direct patient care based on 2015 PSSRU inflated to 2017 costs based on PSSRU inflation indices |
| Health care assistant appointment | £3.83 | Based on PSSRU 2017 band 2 nursing hourly rate and 10 minute appointment |
| 111 calls | £7.00 | NHS Reference costs 2017 |
| A+E attendance | £148.00 | NHS Reference costs 2017 |
| Ambulance call out | £181.00 | NHS Reference costs 2017 |
| Ambulance conveyancing | £248.00 | NHS Reference costs 2017 |
| Hospital admissions | £313 per day | Excess bed day based on NHS Reference costs 2017 |

Sources

PSSRU 2017 [31]

PSSRU 2015 [56]

NHS Reference costs [32]
