# Supplementary material for: Improving primary care Access in Context and Theory (I-ACT trial): a theory-informed randomised cluster feasibility trial using a realist perspective
Source: Trials. 2019 Apr 4;20:193. doi: 10.1186/s13063-019-3299-2 (PMC6449944; doi:10.1186/s13063-019-3299-2)
Supplement: Supplementary file 2 — Table S2. Characteristics of included practices. (DOCX 15 kb) [file 13063_2019_3299_MOESM2_ESM.docx]

**Table S2** Characteristics of included practices.

|  | Practice A | Practice B | Practice C | Practice D |
| --- | --- | --- | --- | --- |
| **Recruitment to trial** | | | | |
| Eligible (n) | 2,408 | 1,156 | 1,244 | 1,188 |
| Invited (n) | 336 | 280 | 238 | 289 |
| Recruited (n) | 18 | 7 | 4 | 5 |
| **Practice characteristics** | | | | |
| Practice population | 12,000-13,000 | 9,000-10,000 | 8,000-9,000 | 8,000-9,000 |
| Approximate catchment area (km^2^) | 88 | 101 | 147 | 152 |
| Staff profile* | 5 GPs, 11 nursing and HCAs staff, 17 admin and receptionist staff | 6 GPs, 7 nursing staff and 16 admin and reception staff | 7 GPs, 7 nursing staff and 11 admin and reception staff | 8 GPs, 6 nursing staff and 8 admin and reception staff |
| Max no. of staff answering calls | 3 | 4 | 3 | 2 |
| **Results of GP Patient survey 2016/17** | | | | |
| Very or fairly easy to get through on the phone (%) | 53 | 81 | 70 | 100 |
| Very or fairly helpful receptionists (%) | 85 | 100 | 100 | 100 |
| Almost always or a lot of the time able to see preferred GP (%) | 51 | 32 | 89 | 77 |
| Able to get appointment | 94 | 92 | 100 | 100 |
| Appointment same day or next day (%) | 56 | 49 | 67 | 58 |
| Very or fairly good overall experience of making an appointment (%) | 74 | 100 | 90 | 100 |
| Definitely or probably recommend surgery (%) | 82 | 91 | 89 | 100 |

*Includes both full-time and part time staff

GP= general practitioner, HCA = health care assistant, n= number
