# Supplementary material for: Improving primary care Access in Context and Theory (I-ACT trial): a theory-informed randomised cluster feasibility trial using a realist perspective
Source: Trials. 2019 Apr 4;20:193. doi: 10.1186/s13063-019-3299-2 (PMC6449944; doi:10.1186/s13063-019-3299-2)
Supplement: Supplementary file 3 — Table S3. Logic model for intervention practices. (DOCX 16 kb) [file 13063_2019_3299_MOESM3_ESM.docx]

**Table S3** Logic model for intervention practices.

| Target population | Assumptions | Practice inputs | Practice activities | Measures | Outputs | Potential outcomes |
| --- | --- | --- | --- | --- | --- | --- |
| Practice A | | | | | | |
| Patients who do not have transport to get to surgery | Patients find it difficult to get to the surgery leading to poor access | Time to meet with community transport provider  Time to add community transport information to signposting  Time to communicate with receptionists | Set up closer contact with community transport provider  Promote community transport provider at surgery | Number of journeys from community transport to and from surgery | Patients will find it easier to get transport to the surgery  Patients will not have to continually redial until they get through to the surgery  Fewer patients will attend the surgery to book an appointment | Patients find it easier to get to the surgery  Patients are more satisfied with the booking system |
| Any patients phoning to book an appointment | Some patients are having to repeatedly dial the surgery because of an engaged telephone line  Some patients may be put off booking an appointment because of the difficulties in the booking system | Funds to install new telephone system | Install new telephone system to stack calls | Data from new telephone system |  |  |
| Practice B | | | | | | |
| Patients who attend surgery with difficulty but don’t have own transport | Some patients can’t get appropriate appointments because of lack of transport or knowledge of transport options | Practice manager and administrator time to discuss with reception team  Practice manager and administrator time to add transport to signposting information | Add transport to signposting template  Add community transport information to information packs | Data from signposting template and possibly a READ code if signposted to community transport | Patients who attend surgery with difficulty but don’t have own transport | Patients supported with transport to get to appointment  Patients supported with transport to get to appointment  Patients with mobility scooters more confident in accessing practice  Patients understand more about the role of receptionists |
| Patients who use the bus | Some patients not able to get appointment because of bus times | Receptionist time to include transport in signposting and be flexible with appointments | Communicate with reception team about using embargoed appointments to allow bus travel | Date and recipients of memos and aide memories sent  New slot type created for embargoed slots moved to fit in with bus times | Patients who use the bus |  |
| Patients with mobility scooters who need charging facilities | Some patients don’t attend with mobility scooters because they don’t have enough battery charge | Receptionist time to facilitate scooter charging and communicate with individual patients | Communicate with reception team about mobility scooter charging  Communicate with individual patients about mobility scooter charging | Date and recipients of memos or aide memories sent  Details of letters sent | Patients with mobility scooters who need charging facilities |  |
| All patients | Patients read the newsletter and practice leaflets | Leaflet about medical receptionists and articles about receptionists in the newsletter | Promoting role of receptionists | Number of leaflets and newsletters distributed | All patients |  |
| Practice C | | | | | | |
| Patients who rely on taxis | Patients find it difficult to book a taxi at certain times of the day | Commitment to prompt a local taxi firm  Administrator time to organise priority hour | Set up formal arrangements with a local taxi firm | Receptionists will record every time a taxi slot is being used for a taxi, possibly with a READ code  Taxi firm will provide number of journeys to and from practice over past 12 months and Jan-June 2018 | More patients are able to book a taxi  Patients have a better understanding of the role of a receptionist  Lower number of unnecessary appointments  Patients diverted to more appropriate services  Increased confidence of receptionists | Patients find it easier to get to the surgery  Patients get better access to the help they need first time  Receptionists more confident in helping patients to the right service |
| Patients who don’t have transport to get to the surgery | Patients may not know about community transport options | Administrator and receptionist time to share information about community transport | Training with local signposting organisation with knowledge about community transport | Number of people attending training |  |  |
| Any patient phoning the surgery | Patients are willing to receive advice from receptionists | Time for practice staff to attend triaging and signpost training  Receptionist time to signpost and triage  Funds to pay for training | Training with external company | Number of people attending training |  |  |
