# Supplementary material for: Improving primary care Access in Context and Theory (I-ACT trial): a theory-informed randomised cluster feasibility trial using a realist perspective
Source: Trials. 2019 Apr 4;20:193. doi: 10.1186/s13063-019-3299-2 (PMC6449944; doi:10.1186/s13063-019-3299-2)
Supplement: Supplementary file 4 — Table S4. Intraclass correlation coefficient. (DOCX 14 kb) [file 13063_2019_3299_MOESM4_ESM.docx]

**Table S4** Intraclass correlation coefficient.

| **Variable** | **ICC (95% CI)** |
| --- | --- |
| Ease of booking an appointment | 0.18 (0.01 to 0.83) |
| Convenience of booking appointment | Not estimated |
| Ability to book appointment | Not estimated |
| Transport options | Not estimated |
| Convenience of transport | Not estimated |
| Ability to get suitable transport | Not estimated |
| EQ5D5L | Not estimated |
| ICECAP-O | 0.67 (0.00 to 0.96) |
| PAM | 0.08 (0.00 to 0.94) |

ICECAP-O = ICEpop CAPability measure for Older people. PAM = Patient Activation Measure, ICC = intraclass correlation coefficient
