# Supplementary material for: Improving primary care Access in Context and Theory (I-ACT trial): a theory-informed randomised cluster feasibility trial using a realist perspective
Source: Trials. 2019 Apr 4;20:193. doi: 10.1186/s13063-019-3299-2 (PMC6449944; doi:10.1186/s13063-019-3299-2)
Supplement: Supplementary file 5 — Table S5. Quality of care at baseline and follow-up for those with complete data. (DOCX 26 kb) [file 13063_2019_3299_MOESM5_ESM.docx]

**Table S5** Quality of care at baseline and follow-up for those with complete data.

|  | | | Practice | | | | | | Intervention  (n=27) | | Usual care  (n=3) | |
| --- | --- | --- | --- | --- | --- | --- | --- | --- | --- | --- | --- | --- |
|  |  |  | A (n=17) | | B (n=5) | | C (n=4) | |  |  |  |  |
|  |  |  | Baseline | Follow-up | Baseline | Follow-up | Baseline | Follow-up | Baseline | Follow-up | Baseline | Follow-up |
| GP | Giving enough time, n (%) | Very poor | 0 (0%) | 0 (0%) | 0 (0%) | 0 (0%) | 0 (0%) | 0 (0%) | 0 (0%) | 0 (0%) | 0 (0%) | 0 (0%) |
|  |  | Poor | 1 (6%) | 0 (0%) | 0 (0%) | 0 (0%) | 0 (0%) | 0 (0%) | 1 (4%) | 0 (0%) | 0 (0%) | 1 (33%) |
|  |  | Neither | 1 (6%) | 2 (12%) | 1 (20%) | 1 (20%) | 0 (0%) | 1 (25%) | 2 (8%) | 4 (15%) | 1 (33%) | 0 (0%) |
|  |  | Good | 6 (35%) | 8 (47%) | 1 (20%) | 0 (0%) | 0 (0%) | 1 (25%) | 7 (27%) | 9 (35%) | 0 (0%) | 1 (33%) |
|  |  | Very good | 9 (53%) | 7 (41%) | 3 (60%) | 4 (80%) | 4 (100%) | 2 (50%) | 16 (62%) | 13 (50%) | 2 (67%) | 1 (33%) |
|  | Listening to you, n (%) | Very poor | 0 (0%) | 0 (0%) | 0 (0%) | 0 (0%) | 0 (0%) | 0 (0%) | 0 (0%) | 0 (0%) | 0 (0%) | 0 (0%) |
|  |  | Poor | 1 (7%) | 0 (0%) | 0 (0%) | 0 (0%) | 0 (0%) | 0 (0%) | 1 (4%) | 0 (0%) | 1 (33%) | 0 (0%) |
|  |  | Neither | 0 (0%) | 0 (0%) | 1 (20%) | 1 (20%) | 0 (0%) | 1 (25%) | 1 (4%) | 2 (8%) | 0 (0%) | 0 (0%) |
|  |  | Good | 5 (33%) | 5 (31%) | 1 (20%) | 0 (0%) | 1 (25%) | 1 (25%) | 7 (29%) | 6 (24%) | 0 (0%) | 2 (67%) |
|  |  | Very good | 9 (60%) | 11 (69%) | 3 (60%) | 4 (80%) | 3 (75%) | 2 (50%) | 15 (63%) | 17 (68%) | 2 (67%) | 1 (33%) |
|  | Explaining tests and treatments, n (%) | Very poor | 0 (0%) | 0 (0%) | 0 (0%) | 0 (0%) | 0 (0%) | 0 (0%) | 0 (0%) | 0 (0%) | 0 (0%) | 0 (0%) |
|  |  | Poor | 1 (7%) | 0 (0%) | 0 (0%) | 0 (0%) | 0 (0%) | 0 (0%) | 1 (5%) | 0 (0%) | 0 (0%) | 0 (0%) |
|  |  | Neither | 1 (7%) | 2 (13%) | 1 (20%) | 1 (20%) | 0 (0%) | 0 (0%) | 2 (9%) | 3 (13%) | 1 (33%) | 0 (0%) |
|  |  | Good | 5 (36%) | 3 (20%) | 1 (20%) | 0 (0%) | 1 (33%) | 1 (33%) | 7 (32%) | 4 (17%) | 0 (0%) | 1 (33%) |
|  |  | Very good | 7 (50%) | 10 (67%) | 3 (60%) | 4 (80%) | 2 (67%) | 2 (67%) | 12 (55%) | 16 (70%) | 2 (67%) | 2 (67%) |
|  | Involving you in decisions, n (%) | Very poor | 1 (8%) | 0 (0%) | 0 (0%) | 0 (0%) | 0 (0%) | 0 (0%) | 1 (5%) | 0 (0%) | 0 (0%) | 0 (0%) |
|  |  | Poor | 0 (0%) | 0 (0%) | 0 (0%) | 0 (0%) | 0 (0%) | 0 (0%) | 0 (0%) | 0 (0%) | 0 (0%) | 0 (0%) |
|  |  | Neither | 2 (15%) | 2 (13%) | 1 (20%) | 0 (0%) | 0 (0%) | 0 (0%) | 3 (14%) | 2 (9%) | 0 (0%) | 0 (0%) |
|  |  | Good | 2 (15%) | 5 (33%) | 1 (20%) | 0 (0%) | 1 (25%) | 2 (50%) | 4 (18%) | 7 (30%) |  | 0 (0%) |
|  |  | Very good | 8 (62%) | 8 (53%) | 3 (60%) | 4 (100%) | 3 (75%) | 2 (50%) | 14 (64%) | 14 (61%) | 3 (100%) | 2 (100%) |
|  | Treating you with care and concern, n (%) | Very poor | 0 (0%) | 0 (0%) | 0 (0%) | 0 (0%) | 0 (0%) | 0 (0%) | 0 (0%) | 0 (0%) | 0 (0%) | 0 (0%) |
|  |  | Poor | 0 (0%) | 0 (0%) | 0 (0%) | 0 (0%) | 0 (0%) | 0 (0%) | 0 (0%) | 0 (0%) | 0 (0%) | 0 (0%) |
|  |  | Neither | 0 (0%) | 1 (6%) | 1 (20%) | 1 (20%) | 0 (0%) | 0 (0%) | 1 (4%) | 2 (8%) | 0 (0%) | 0 (0%) |
|  |  | Good | 7 (44%) | 3 (19%) | 1 (20%) | 0 (0%) | 1 (25%) | 2 (50%) | 9 (36%) | 5 (20%) | 0 (0%) | 0 (0%) |
|  |  | Very good | 9 (56%) | 12 (75%) | 3 (60%) | 4 (80%) | 3 (75%) | 2 (50%) | 15 (60%) | 18 (72%) | 3 (100%) | 3 (100%) |
|  | Confidence and trust, n (%) | No, not at all | 0 (0%) | 1 (6%) | 0 (0%) | 0 (0%) | 0 (0%) | 0 (0%) | 0 (0%) | 1 (4%) | 0 (0%) | 0 (0%) |
|  |  | Yes, to some extent | 7 (44%) | 5 (29%) | 1 (20%) | 1 (20%) | 1 (25%) | 1 (25%) | 9 (36%) | 7 (27%) | 1 (33%) | 0 (0%) |
|  |  | Yes, definitely | 9 (56%) | 11 (65%) | 4 (80%) | 4 (80%) | 3 (75%) | 3 (75%) | 16 (64%) | 18 (69%) | 2 (67%) | 3 (100%) |
| Nurse | Giving enough time, n (%) | Very poor | 0 (0%) | 0 (0%) | 0 (0%) | 0 (0%) | 0 (0%) | 0 (0%) | 0 (0%) | 0 (0%) | 0 (0%) | 0 (0%) |
|  |  | Poor | 1 (6%) | 0 (0%) | 0 (0%) | 0 (0%) | 0 (0%) | 0 (0%) | 1 (4%) | 0 (0%) | 0 (0%) | 0 (0%) |
|  |  | Neither | 0 (0%) | 0 (0%) | 0 (0%) | 1 (20%) | 0 (0%) | 0 (0%) | 0 (0%) | 1 (4%) | 0 (0%) | 0 (0%) |
|  |  | Good | 6 (35%) | 8 (50%) | 3 (60%) | 1 (20%) | 1 (25%) | 2 (50%) | 10 (38%) | 11 (44%) | 1 (33%) | 2 (67%) |
|  |  | Very good | 10 (59%) | 8 (50%) | 2 (40%) | 3 (60%) | 3 (75%) | 2 (50%) | 15 (58%) | 13 (52%) | 2 (67%) | 1 (33%) |
|  | Listening to you, n (%) | Very poor | 0 (0%) | 0 (0%) | 0 (0%) | 0 (0%) | 0 (0%) | 0 (0%) | 0 (0%) | 0 (0%) | 0 (0%) | 0 (0%) |
|  |  | Poor | 0 (0%) | 0 (0%) | 0 (0%) | 0 (0%) | 0 (0%) | 0 (0%) | 0 (0%) | 0 (0%) | 0 (0%) | 0 (0%) |
|  |  | Neither | 2 (13%) | 0 (0%) | 1 (20%) | 2 (40%) | 0 (0%) | 0 (0%) | 3 (12%) | 2 (8%) | 0 (0%) | 0 (0%) |
|  |  | Good | 5 (31%) | 10 (63%) | 2 (40%) | 0 (0%) | 1 (25%) | 2 (50%) | 8 (32%) | 12 (48%) | 1 (33%) | 1 (33%) |
|  |  | Very good | 9 (56%) | 6 (38%) | 2 (40%) | 3 (60%) | 3 (75%) | 2 (50%) | 14 (56%) | 11 (44%) | 2 (67%) | 2 (67%) |
|  | Explaining tests and treatments, n (%) | Very poor | 1 (6%) | 0 (0%) | 0 (0%) | 0 (0%) | 0 (0%) | 0 (0%) | 1 (4%) | 0 (0%) | 0 (0%) | 0 (0%) |
|  |  | Poor | 0 (0%) | 0 (0%) | 0 (0%) | 0 (0%) | 0 (0%) | 0 (0%) | 0 (0%) | 0 (0%) | 0 (0%) | 0 (0%) |
|  |  | Neither | 2 (12%) | 2 (13%) | 1 (20%) | 1 (25%) | 0 (0%) | 0 (0%) | 3 (12%) | 3 (13%) | 0 (0%) | 0 (0%) |
|  |  | Good | 5 (29%) | 6 (40%) | 2 (40%) | 0 (0%) | 1 (25%) | 2 (50%) | 8 (31%) | 8 (35%) | 1 (50%) | 1 (33%) |
|  |  | Very good | 9 (53%) | 7 (47%) | 2 (40%) | 3 (75%) | 3 (75%) | 2 (50%) | 14 (54%) | 12 (52%) | 1 (50%) | 2 (67%) |
|  | Involving you in decisions, n (%) | Very poor | 0 (0%) | 0 (0%) | 0 (0%) | 0 (0%) | 0 (0%) | 0 (0%) | 0 (0%) | 0 (0%) | 0 (0%) | 0 (0%) |
|  |  | Poor | 0 (0%) | 1 (9%) | 0 (0%) | 0 (0%) | 0 (0%) | 0 (0%) | 0 (0%) | 1 (5%) | 0 (0%) | 0 (0%) |
|  |  | Neither | 2 (13%) | 2 (18%) | 1 (20%) | 2 (40%) | 0 (0%) | 0 (0%) | 3 (13%) | 4 (20%) | 0 (0%) | 0 (0%) |
|  |  | Good | 5 (33%) | 4 (36%) | 2 (40%) | 0 (0%) | 1 (25%) | 2 (50%) | 8 (33%) | 6 (30%) | 1 (50%) | 0 (0%) |
|  |  | Very good | 8 (53%) | 4 (36%) | 2 (40%) | 3 (60%) | 3 (75%) | 2 (50%) | 13 (54%) | 9 (45%) | 1 (50%) | 2 (100%) |
|  | Treating you with care and concern, n (%) | Very poor | 0 (0%) | 0 (0%) | 0 (0%) | 0 (0%) | 0 (0%) | 0 (0%) | 0 (0%) | 0 (0%) | 0 (0%) | 0 (0%) |
|  |  | Poor | 0 (0%) | 0 (0%) | 0 (0%) | 0 (0%) | 0 (0%) | 0 (0%) | 0 (0%) | 0 (0%) | 0 (0%) | 0 (0%) |
|  |  | Neither | 0 (0%) | 1 (6%) | 0 (0%) | 2 (40%) | 0 (0%) | 0 (0%) | 0 (0%) | 3 (12%) | 0 (0%) | 0 (0%) |
|  |  | Good | 6 (40%) | 6 (38%) | 3 (60%) | 0 (0%) | 1 (25%) | 2 (50%) | 10 (42%) | 8 (32%) | 1 (33%) | 1 (33%) |
|  |  | Very good | 9 (60%) | 9 (56%) | 2 (40%) | 3 (60%) | 3 (75%) | 2 (50%) | 14 (58%) | 14 (56%) | 2 (67%) | 2 (67%) |
|  | Confidence and trust, n (%) | No, not at all | 0 (0%) | 1 (6%) | 0 (0%) | 0 (0%) | 0 (0%) | 0 (0%) | 0 (0%) | 1 (4%) | 0 (0%) | 0 (0%) |
|  |  | Yes, to some extent | 3 (18%) | 2 (12%) | 2 (40%) | 2 (40%) | 0 (0%) | 0 (0%) | 5 (19%) | 4 (15%) | 0 (0%) | 0 (0%) |
|  |  | Yes, definitely | 14 (82%) | 14 (82%) | 3 (60%) | 3 (60%) | 4 (100%) | 4 (100%) | 21 (81%) | 21 (81%) | 3 (100%) | 3 (100%) |
| Recommend surgery, n (%) | | No, definitely not | 0 (0%) | 0 (0%) | 0 (0%) | 0 (0%) | 0 (0%) | 0 (0%) | 0 (0%) | 0 (0%) | 0 (0%) | 0 (0%) |
|  |  | No, probably not | 0 (0%) | 0 (0%) | 0 (0%) | 0 (0%) | 0 (0%) | 0 (0%) | 0 (0%) | 0 (0%) | 0 (0%) | 0 (0%) |
|  |  | Not sure | 1 (6%) | 2 (12%) | 0 (0%) | 0 (0%) | 0 (0%) | 1 (25%) | 1 (4%) | 3 (12%) | 0 (0%) | 0 (0%) |
|  |  | Yes, probably | 7 (39%) | 6 (35%) | 1 (20%) | 1 (20%) | 0 (0%) | 0 (0%) | 8 (30%) | 7 (27%) | 0 (0%) | 0 (0%) |
|  |  | Yes, definitely | 10 (56%) | 9 (53%) | 4 (80%) | 4 (80%) | 4 (100%) | 3 (75%) | 18 (67%) | 16 (62%) | 3 (100%) | 3 (100%) |

N.B. ‘Don’t know’ or ‘Not applicable’ responses have not been included.
