# Supplementary material for: Improving primary care Access in Context and Theory (I-ACT trial): a theory-informed randomised cluster feasibility trial using a realist perspective
Source: Trials. 2019 Apr 4;20:193. doi: 10.1186/s13063-019-3299-2 (PMC6449944; doi:10.1186/s13063-019-3299-2)
Supplement: Supplementary file 6 — Table S6. Resource use activity and associated costs. (DOCX 23 kb) [file 13063_2019_3299_MOESM6_ESM.docx]

**Table S6** Resource use activity and associated costs.

| Resource use | | Practice | | | | | | Intervention total  (n=29) | | Usual care  (n=5) | |
| --- | --- | --- | --- | --- | --- | --- | --- | --- | --- | --- | --- |
|  |  | A (n=18) | | B (n=7) | | C (n=4) | |  |  |  |  |
|  |  | n | £ | n | £ | n | £ | n | £ | n | £ |
| Any primary care contact | Prev 6 months | 99 | £1,420 | 14 | £367 | 26 | £417 | 139 | £2,203 | 26 | £438 |
|  | Follow-up 6 months | 93 | £1,951 | 30 | £1,145 | 28 | £369 | 151 | £3,464 | 26 | £715 |
| GP surgery visit | Prev 6 months | 30 | £930 | 7 | £217 | 5 | £155 | 42 | £1,302 | 8 | £248 |
|  | Follow-up 6 months | 45 | £1,395 | 9 | £588 | 2 | £131 | 56 | £2,114 | 9 | £279 |
| GP telephone | Prev 6 months | 3 | £73 | 0 | £0 | 0 | £0 | 3 | £73 | 0 | £0 |
|  | Follow-up 6 months | 3 | £73 | 3 | £73 | 0 | £0 | 6 | £146 | 1 | £24 |
| GP home visit | Prev 6 months | 0 | £0 | 2 | £131 | 0 | £0 | 2 | £131 | 0 | £0 |
|  | Follow-up 6 months | 0 | £0 | 6 | £392 | 0 | £0 | 6 | £392 | 5 | £327 |
| Nurse surgery visit | Prev 6 months | 19 | £237 | 0 | £0 | 21 | £262 | 40 | £499 | 14 | £175 |
|  | Follow-up 6 months | 36 | £449 | 5 | £62 | 16 | £199 | 57 | £711 | 4 | £50 |
| Nurse telephone | Prev 6 months | 0 | £0 | 0 | £0 | 0 | £0 | 0 | £0 | 0 | £0 |
|  | Follow-up 6 months | 0 | £0 | 2 | £10 | 0 | £0 | 2 | £10 | 7 | £35 |
| HCA surgery visit | Prev 6 months | 47 | £180 | 5 | £19 | 0 | £0 | 52 | £199 | 4 | £15 |
|  | Follow-up 6 months | 9 | £34 | 5 | £19 | 10 | £38 | 24 | £92 | 0 | £0 |
| Any unplanned secondary care contact | Prev 6 months | 8 | £14,220 | 3 | £3,607 | 0 | £0 | 11 | £17,827 | 1 | £148 |
|  | Follow-up 6 months | 11 | £533 | 8 | £2,492 | 0 | £0 | 19 | £3,025 | 2 | £4,850 |
| A+E visits | Prev 6 months | 5 | £740 | 2 | £296 | 0 | £0 | 7 | £1,036 | 1 | £148 |
|  | Follow-up 6 months | 2 | £296 | 2 | £296 | 0 | £0 | 4 | £592 | 1 | £148 |
| Out of hours calls | Prev 6 months | 3 | £21 | 0 | £0 | 0 | £0 | 3 | £21 | 0 | £0 |
|  | Follow-up 6 months | 8 | £56 | 3 | £21 | 0 | £0 | 11 | £77 | 1 | £7 |
| Ambulance call out | Prev 6 months | 0 | £0 | 1 | £181 | 0 | £0 | 1 | £181 | 0 | £0 |
|  | Follow-up 6 months | 1 | £181 | 2 | £362 | 0 | £0 | 3 | £543 | 0 | £0 |
| Ambulance conveyancing | Prev 6 months | 0 | £0 | 0 | £0 | 0 | £0 | 0 | £0 | 0 | £0 |
|  | Follow-up 6 months | 0 | £0 | 1 | £248 | 0 | £0 | 1 | £248 | 0 | £0 |
| Unplanned hospital admissions n, days | Prev 6 months | 3, 43 | £13,459 | 2, 10 | £3,130 | 0,0 | £0 | 5, 53 | £16,589 | 0,0 | £0 |
|  | Follow-up 6 months | 0,0 | £0 | 4, 5 | £1,565 | 0,0 | £0 | 4, 5 | £1,565 | 2, 15 | £4,695 |
